# Effect of activated carbon microstructure and adsorption mechanism on the efficient removal of chlorophyll a and chlorophyll b from *Andrographis paniculata* extract

**DOI:** 10.1038/s41598-023-42011-6

**Published:** 2023-12-11

**Authors:** Di Liang, Bao-yu Ji, Yun Wang, Xia Li, Wen-Yuan Gao

**Affiliations:** 1 Tianjin Key Laboratory for Advanced Drug Delivery & High-Efficiency, School of Pharmaceutical Science and Technology, Tianjin University, No. 92, Weijin Road, Nankai District, Tianjin, 300193 China; 2 College of Pharmacy, Henan University of Chinese Medicine, Zhengzhou, 450046 Henan China; 3 College of Pharmacy, Qinghai Minzu University, Qinghai, 810007 China

**Keywords:** Plant sciences, Materials science

## Abstract

In order to reveal the effect of activated carbon (AC) properties on the adsorption of chlorophyll a (Chl a) and chlorophyll b (Chl b) in *Andrographis paniculata* extract, four commercial activated carbons were first tested and characterized. The results showed that activated carbon 1 (AC1) had the best surface area, pore structure and adsorption capacity. Therefore, adsorption isotherms, adsorption kinetics and adsorption mechanism were further carried out on AC1. The application of Langmuir model (*R*^2^ > 0.978) and Freundlich model (*R*^2^ > 0.977) indicated that the adsorption process of Chl a and Chl b on AC1 may be a complex adsorption process of single-layer and multilayer adsorption. The adsorption kinetics indicated that the pseudo-second-order kinetic model (*R*^2^ > 0.999) was dominant and was mainly chemisorption. The intra-particle diffusion model (*R*^2^ > 0.937) shows that the intra-particle diffusion is the rate-limiting step. The decrease of adsorption of AC1 to Chl a and Chl b due to the oxidation of acrylic acid proves the importance of π–π interaction.

## Introduction

In recent years, natural active monomeric compounds isolated from herbal medicines have become the best choice to reduce the side effects of preparations and develop high value-added products^1,2^. Andrographolide, the main active ingredient of *Andrographis paniculata* (*A. paniculata*), is already marketed in China and Southeast Asian known as a natural antibiotic used to treat sore throats, chills, influenza and other ailments^3^. The preparation of andrographolide from *A. paniculata* can be divided into extraction, decolorization, concentration, impurity removal and crystallization. The extract of *A. paniculata* usually contains a lot of chlorophyll, mainly chlorophyll a (Chl a) and chlorophyll b (Chl b). If the extract was concentrated directly, dark green thick lump will be produced affecting the subsequent separation and purification process. In addition, during the crystallization process, little chlorophyll causes the crystals of andrographolide to appear green, which affects the purity, value and biological activity of the product. Therefore, decolorization is an extremely important unit operation.

So far, many methods such as membrane treatment, adsorption, photolysis, pyrolysis and oxidation have been used to remove pollutants from solutions^4^. However, most of these technologies are environmentally destructive, inefficient, operationally limited and cost prohibitive^5^. In the above methods, adsorption technology, especially activated carbon (AC) adsorption technology, is considered as the best decolorization method for its advantages of simple operation, low cost and good decolorization effect, and it has become the standard operation of decolorization in many countries^6^. However, it was found that AC produced by different manufacturers, even if they had similar adsorption values of iodine and methylene blue (usually used to evaluate the adsorption performance of AC), their removal rates of Chl a and Chl b were very different. It is often time-consuming and costly for enterprises to select appropriate AC to remove Chl a and Chl b. Therefore, it is urgent to reveal the intrinsic properties of AC with excellent Chl a and Chl b adsorption.

At present, most researches on AC mainly focus on dyes and metal ions, and there are few researches on the removal of Chl a and Chl b. from herbal extracts with AC microstructure and adsorption mechanism^7,8^. More studies have shown that the adsorption capacity of AC is related to its specific surface area, pore size, surface charge, pore size distribution and surface function^9–11^. Reasonable high specific surface area or micropore volume can provide more adsorption sites in favor of higher adsorption performance^12^. Pore size must be large enough to accommodate the size of the adsorbed molecule. Micropores (0–2 nm) mainly adsorbed low molecular weight compounds, mesoporous (2–50 nm) mainly adsorbed large molecular weight organic compounds, macropores (> 50 nm) suitable for adsorption of microorganisms^13^. Saleem et al.^14^ showed that dyes and humic acids (1.5 ~ 3.0 nm) were more likely to adsorb in mesoporous pores. For adsorbents with molecular diameters of about 0.5 nm, microporous AC is usually sought as an adsorbent. Appropriate surface functional groups will produce a positively, neutrally or negatively charged surface, which may improve the adsorption capacity of AC by improving the electrostatic attraction between AC and the adsorbent^15^. In our previous review^16^, we also found that the adsorption capacity and properties of AC are fantastically related to its activation methods, activation conditions and raw material sources. Changes in the type of activation, type of chemical activator, impregnation time, carbonization temperature or chemical composition of raw materials will significantly affect the structure of AC. Understanding the relationship between the structural characteristics of AC and the decolorization effect, as well as the relationship between the adsorption mechanism and the decolorization effect, are helpful for AC manufacturers to improve the interaction between AC and Chl a and Chl b through modification, thus improving the adsorption performance.

Therefore, in this study, four commercial ACs were first tested and characterized hoping to reveal the influence of the microstructure of AC on the removal of Chl a and Chl b. Adsorption isotherm, adsorption kinetics and adsorption mechanism have important guiding significance to decolorization process and AC process optimization. So, through an equilibrium adsorption experiment, adsorption isotherm, adsorption kinetics and adsorption mechanism of AC with the best decolorization effect were studied.

## Materials and methods

### Chemicals

Analytic grade ethanol, HNO_3_, acrylic acid, HCl and NaOH were provided by Jiangtian Chemical Technology Co. (Tianjin). Standards Chl a (purity 85%) and Chl b (purity 90%) were purchased from Meryer Chemical Reagents LTD. (Shanghai). *A. paniculata* was purchased from Wanggui Herbal Medicine Base (Guangxi) and the material was extracted by A type AS20500AT ultrasonic cleaner (Tianjin) with ethanol concentration of 90% (V/V), solid–liquid ratio of 1:10, ultrasonic power of 330W, ultrasonic time of 60 min, ultrasonic temperature of 50 °C, and the combined extraction solution of two extraction times was adsorption solution. Plants are harvested in their mature stage and it was identified as true by Wenyuan Gao, a medicinal botanist. The field study of this experiment is in accordance with relevant regulations and guidelines and carried out in accordance with relevant regulations.

### Preparation and characterization of activated carbon

Four kinds of powdered AC were purchased from Nanpingyuanli Activated Carbon Company (Jiangxi) (AC1), Junfan Activated Carbon Company (Shanghai) (AC2), Mulinsen Activated Carbon Company (Jiangsu) (AC3) and Hanyan Activated Carbon Company (Guangdong) (AC4), respectively. Four ACs were characterized by SEM, FTIR, Brunauer–Emmett–Teller (BET) and XRD. For the SEM test, a Nanosem 430 environmental scanning electron microscope (FEI) was used. The samples are pretreated by gold spraying. The functional groups on the AC surface were detected by FTIR (Bruker); About 1.0 mg of AC powder was used by mixing with 100 mg KBr for FTIR. N_2_ adsorption–desorption isotherms were performed by Autosorb-iQ2-XR Quanta chrome (USA) at 77 K to characterize the physical properties of the four ACs. Based on the N_2_ adsorption–desorption isotherm, the specific surface areas of four ACs were estimated using the BET equation^17^. In addition, the Barrette-Joynere-Halenda (BJH) method^18^ was used to obtain the cumulative pore volume, average pore size and pore size distribution. X-ray diffraction analysis was performed to determine the crystallinity or amorphous properties of four ACs. The analysis was performed with the Smart Lab Intelligent X-ray diffraction system, and the diffraction angles (2θ) ranged from 5° to 80°.

### Batch adsorption experiments

Batch adsorption experiments were implemented in a conical flask containing quantitative *A. paniculata* extract solution and adsorbent. All the adsorption experiments were conducted in parallel for 3 times at 150 rpm min^−1^. After the experiment, the samples were separated by filtration membrane (0.45 μm), the contents of Chl a and Chl b in the supernatant were recorded.

The four ACs (about 120 mg) were mixed with the extract (150 ml) at 35, 45 and 55 °C, respectively, for 2 h to screen out the AC with the best adsorption effect. Based on previously determined adsorption kinetics (data not shown), these times are sufficient to achieve apparent adsorption equilibrium where the rate of adsorption equals the rate of desorption. The selected AC with the best adsorption effect was further explored for adsorption isotherm, adsorption kinetics and adsorption mechanism.

In order to investigate the adsorption isotherms, the adsorption isotherm experiments were studied at 35 °C, 45 °C and 55 °C when the equilibrium adsorption time was 70 min and the amount of AC was 40, 60, 80, 100, 120 and 140 mg, respectively. In order to investigate the adsorption kinetic, the adsorption kinetic experiments were studied at 35 °C, 45 °C and 55 °C when the amount of AC was 120 mg and the adsorption time was 5, 10, 20, 30, 40, 50, 60 and 70 min, respectively. In order to study whether the adsorption is reversible, the used AC (120 mg) was placed in a clean 90% ethanol solution at 35, 45, 55 °C and stirred for 2 h. The presence of any π–π interaction was determined by acrylic oxidation experiments^19^. Dip about 1.0 g of AC into 100 ml of DD water containing 20 ml of acrylic. The mixture was stirred at 200 rpm for 1 h and then transferred to a teflon-lined autoclave (200 ml) for hydrothermal treatment of 24 h at 190 °C, the black solid oxygenate was separated from the solution and washed with DD water until the pH of the filtrate reaches a constant value. Dry the black solid at 60 °C for 12 h for reserve use^20^. 120 mg of the above black solid and unoxidized AC were added into 150 ml of *A. paniculata* extract, and stirred at 55 °C for 2 h.

### Chlorophyll a and chlorophyll b analysis

Chl a and Chl b have maximum absorption wavelengths at 663 nm and 645 nm, so the absorbance of Chl a and Chl b is determined in UV (Agilent-1100 series, USA) at 663 nm and 645 nm. The concentrations of Chl a and Chl b were determined by the standard curve with *R*^2^ of 0.9999.

### Data analysis

Equilibrium adsorption capacity *q*_*e*_ of Chl a and Chl b is calculated by the following mass balance equation:

1 $${q}_{e}=({C}_{0}-{C}_{\mathrm{e}})\frac{V}{\mathrm{W}}$$

where *q*_*e*_/(mg g^−1^) is equilibrium adsorption capacity; *C*_*e*_/(mg L^−1^) is equilibrium mass concentration; *C*_*0*_/(mg L^−1^) is Chl a or Chl b concentration at zero time; *W*/(g) is the amount of AC; *V*/(L) is the volume of solution.

Langmuir model and Freundlich model were used to study adsorption isotherm:

2 $${q}_{t}=\frac{{q}_{\mathrm{max}}{K}_{\mathrm{L}}{C}_{\mathrm{e}}}{\left(1+{K}_{\mathrm{L}}\right){C}_{\mathrm{e}}}(\mathrm{Langmuir model})$$

3 $${q}_{t}={K}_{\mathrm{F}}{{C}_{\mathrm{e}}}^{1/n} \quad \quad (\mathrm{Freundlich \, model})$$

where *q*_*max*_/(mg g^−1^) is the maximum single-layer adsorption capacity; *q*_*t*_/(mg g^−1^) is the amount of adsorption at time *t*; *K*_*L*_/(mg L^−1^) is the adsorption constant. 1/*n* is the heterogeneous factor, n represents the degree of isotherm deviation from linearity (1/*n* is between 0.1 and 1.0, indicating that the adsorption process is easy to proceed.)

The thermodynamic parameter free energy (∆*G*_*0*_) can be calculated by the following formula:

4 $${\Delta G}^{0}=-RTLn{K}_{C}$$

The standard adsorption enthalpy (*∆H*_*0*_) and the standard adsorption entropy (*∆S*_*0*_) can be obtained by Van't Hoff's formula:

5 $$Ln{K}_{C} =\frac{{\Delta S}^{0}}{R}-\frac{{\Delta H}^{0}}{RT}$$

where *R* is the gas molar constant, *R* = 8.31 J mol K^−1^; *T/*(K) is the absolute temperature; *Kc* is the distribution coefficient, ${K}_{C} =\frac{{C}_{Ae}}{{C}_{e}}$, *C*_*Ae*_/(mg L^−1^) is the mass concentration of adsorbed substances and *C*_*e*_ is the mass concentration of the remaining substance.

Adsorption kinetics of the AC was studied by the pseudo-first-order, pseudo-second-order and intra-particle diffusion kinetic models.

7 $${\mathrm{log}({q}_{e}-q}_{t})=\mathrm{log}\left({q}_{e}\right)-\frac{{K}_{1}t}{2.303} \quad \quad (\text{pseudo-first-order model})$$

8 $$\frac{t}{{q}_{t}}=\frac{1}{{k}_{2}{q}_{e}^{2}}+\frac{t}{{q}_{e}}\quad \quad (\text{pseudo-second-order model})$$

9 $${q}_{t}={k}_{p}{t}^{0.5}+C\quad \quad (\text{intra-particle diffusion kinetic model})$$

where *k*_*1*_/(min^−1^) and *k*_*2*_/(g mg^−1^ min^−1^) are the first order kinetic and the second order kinetic adsorption constants, and *k*_*p*_/(mg g^−1^ min^−0.5^) is the intra-particle diffusion rate constant, and *C* is constant which reflects the boundary layer effect.

## Results and discussion

### Adsorption capacity of four activated carbons

**Figure 1:**
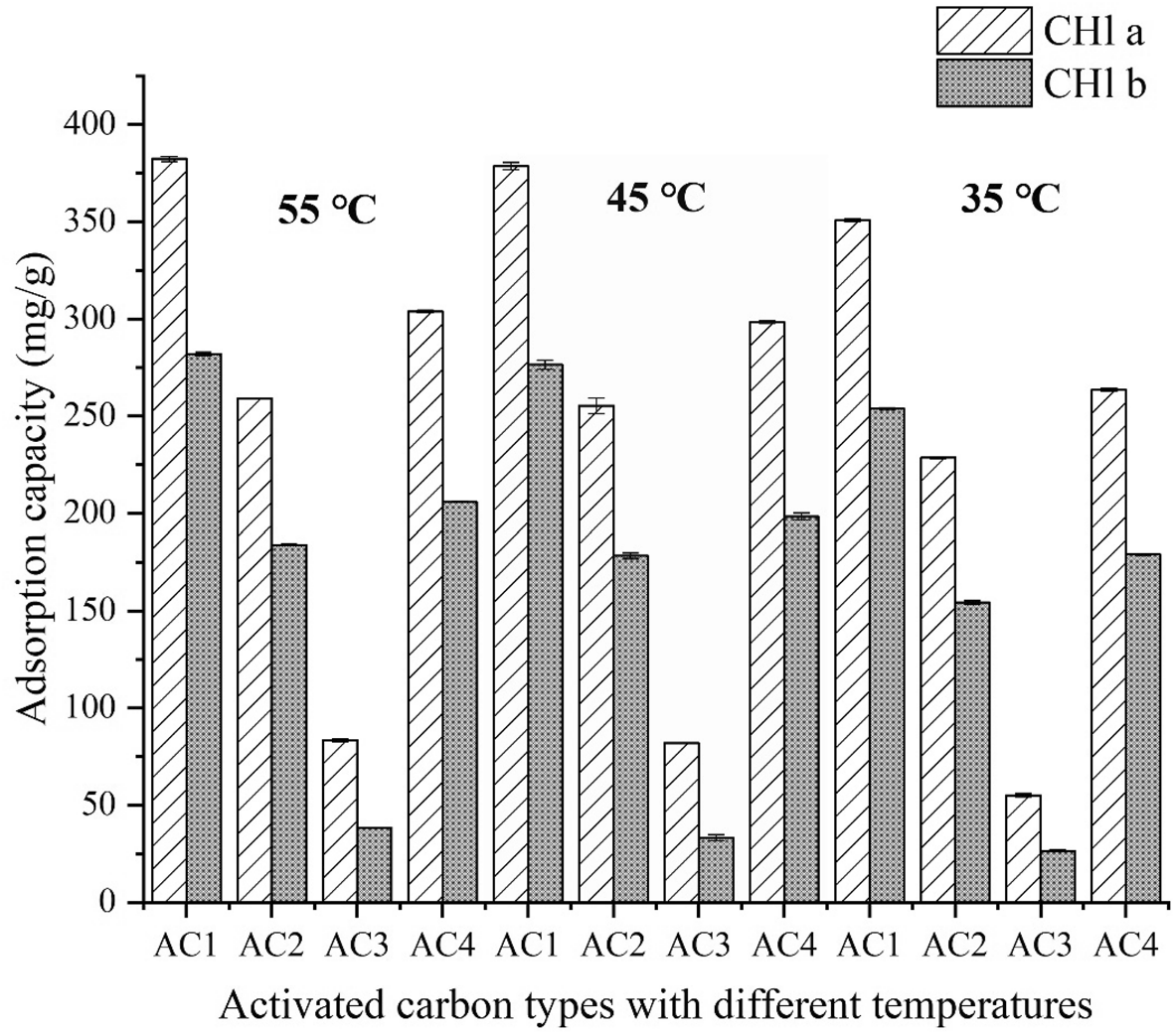
Adsorption capacity of four activated carbons for chlorophyll a and chlorophyll b at 35, 45 and 55 °C.

Chl a and chl b differ in structure only in the additional groups on pyrrole ring II: the former is a methyl group, the latter is a formaldehyde group. Due to the extremely similar structure, if the same AC had a good adsorption capacity for chl a, its adsorption effect of chlorophyll b was also excellent. But the adsorption effect of different AC on the two adsorbed substances is greatly different. Four kinds of ACs adsorption effect is: AC1 > AC4 > AC2 > AC3 (Fig. 1), which shows that different manufacturers of AC for the same target adsorption capacity does have differences.

### Activated carbon characteristics

**Figure 2:**
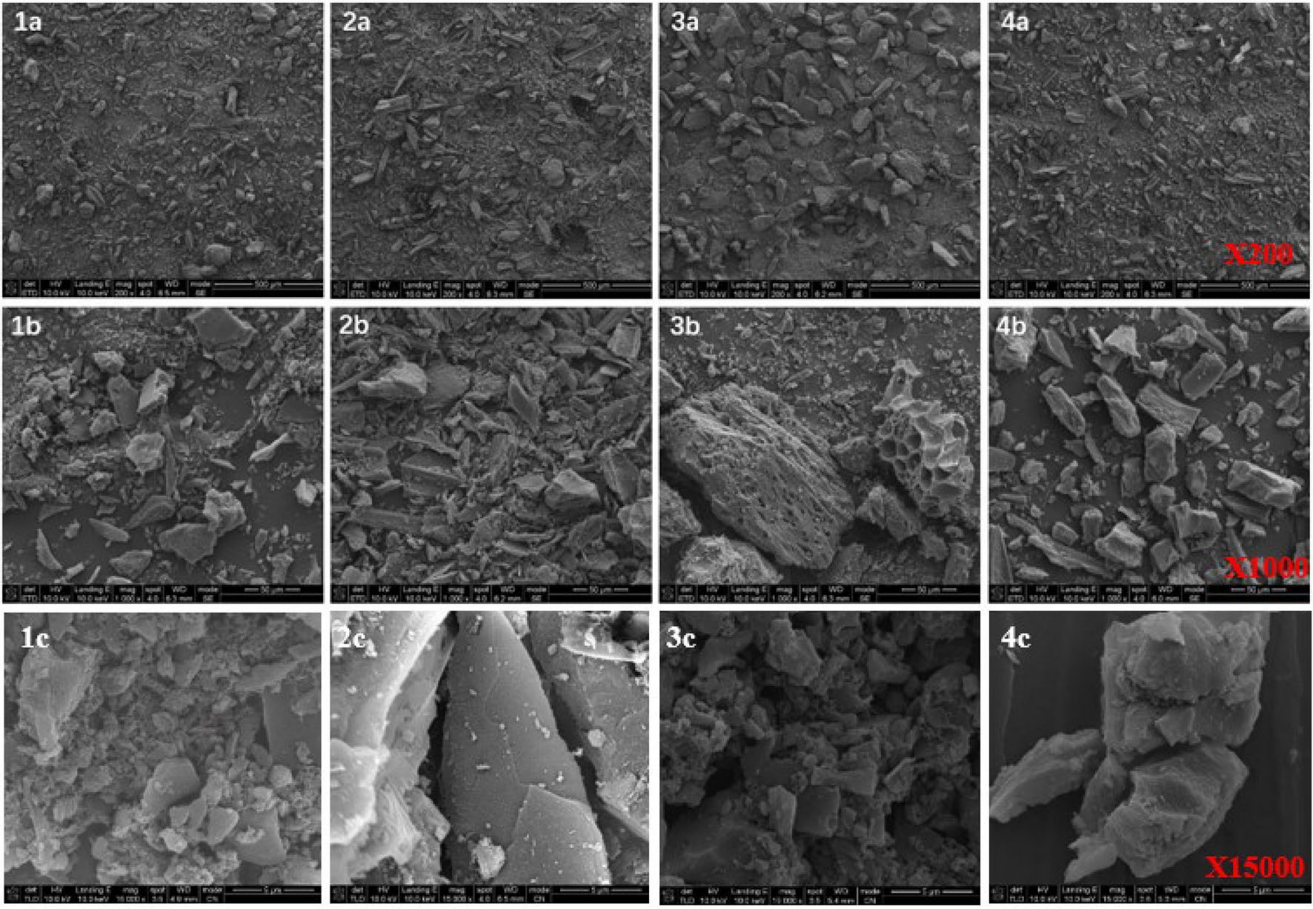
The scanning electron micrographs four activated carbons.

Figure 2 shows the SEM images of the four ACs magnification by 200, 1000 and 15,000 times respectively. All four ACs have wrinkles and depressions on their surfaces, which create favorable conditions for providing a large surface area. Under the 1000 magnification, the particle size of AC3 was observed to be significantly larger than the other three, which indicated that the total specific surface area of AC3 may not be large, even if the surface of AC3 is honeycomb, which is thought to increase the total surface area of ACs. Under the 15,000 magnification, all four activated carbons have dense pores on their surfaces, and the mesoporous and microporous of ACs cannot be identified.

**Figure 3:**
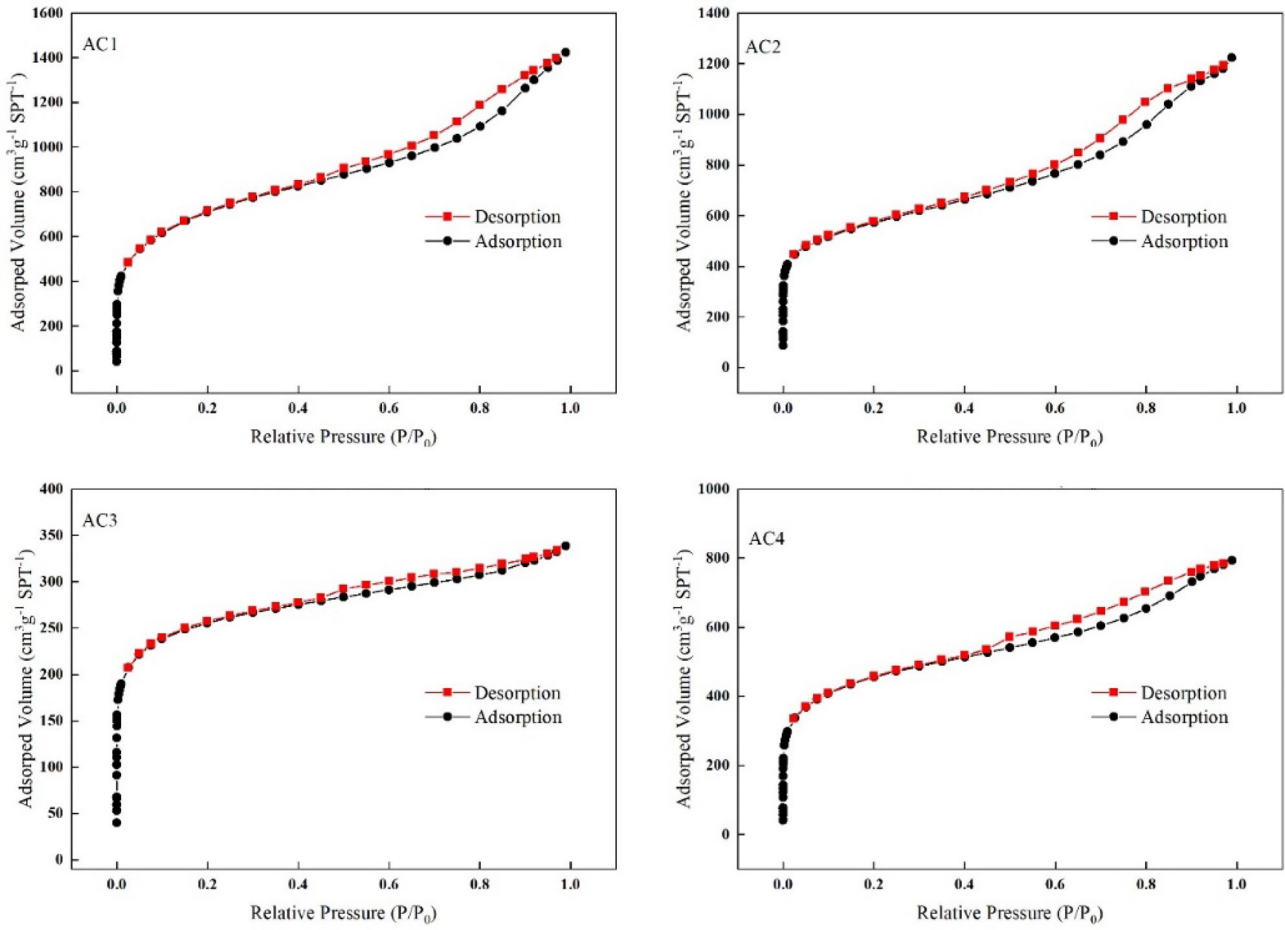
N_2_ adsorption–desorption isotherms of four activated carbons.

As shown in Fig. 3, the N_2_ adsorption–desorption isotherms of the four ACs are similar to the type IV isotherms classified by the International Union of Purity and Applied Chemistry (IUPAC)^21^. All four N_2_ adsorption–desorption isotherms showed H4 hysteresis loops composed of type I and type II isotherms with significantly adsorption capacity at the low end of P/P_0_, which suggested that the adsorption mechanism begins with the formation of single or multiple layers on the surface of AC, followed by pore condensation. Table 1 lists the calculated parameters of total specific surface area (S_BET_), micropore specific surface area (S_Mic_), total pore volume (V_Tot_), micropore volume (V_Mic_) and average pore diameter of the four ACs.

**Table 1:** Structural parameters of four activated carbons.

Sample	S_BET_	S_Mic_		V_Tot_	V_Mic_		S_Ave_
	m^2^ g^−1^	m^2^ g^−1^	%	cc g^−1^	cc g^−1^	%	nm
AC1	2525.68	1995.71	79.02	2.21	1.01	45.69	3.49
AC2	2058.83	1459.78	70.90	1.90	0.70	37.18	3.67
AC3	948.23	865.14	91.24	0.53	0.38	72.57	2.21
AC4	1639.88	1325.72	80.84	1.23	0.62	50.81	3.00

*S*_*BET*_ Total specific surface area, *S*_*Mic*_ Micropore specific surface area, *V*_*Tot*_ Total pore volume, *V*_*Mic*_ Micropore volume, *S*_*Ave*_ Average pore size.

It has been reported that materials with large surface area and narrow pore distribution have more adsorption sites and higher adsorption energy, which has a significant influence on their adsorption behavior^22^. The S_BET_ the four ACs from large to small is: AC1 (2525.68 m^2^ g^−1^) > AC2 (2058.83 m^2^ g^−1^) > AC4 (1639.88 m^2^ g^−1^) > AC3 (948.23 m^2^ g^−1^). The adsorption effect of four ACs was found to be AC1 > AC4 > AC2 > AC3. S_Mic_ accounts for 70 ~ 90% of S_BET_. AC1 has the best adsorption effect with the largest S_BET_, while AC3 has the worst adsorption effect with the smallest S_BET_ in four ACs; The S_BET_ of AC2 is significantly greater than that of AC4, but its adsorption performance for Chl a and Chl b is inferior to AC4, which indicates that the S_BET_ may play a major role in the adsorption process, but not a decisive role.

**Figure 4:**
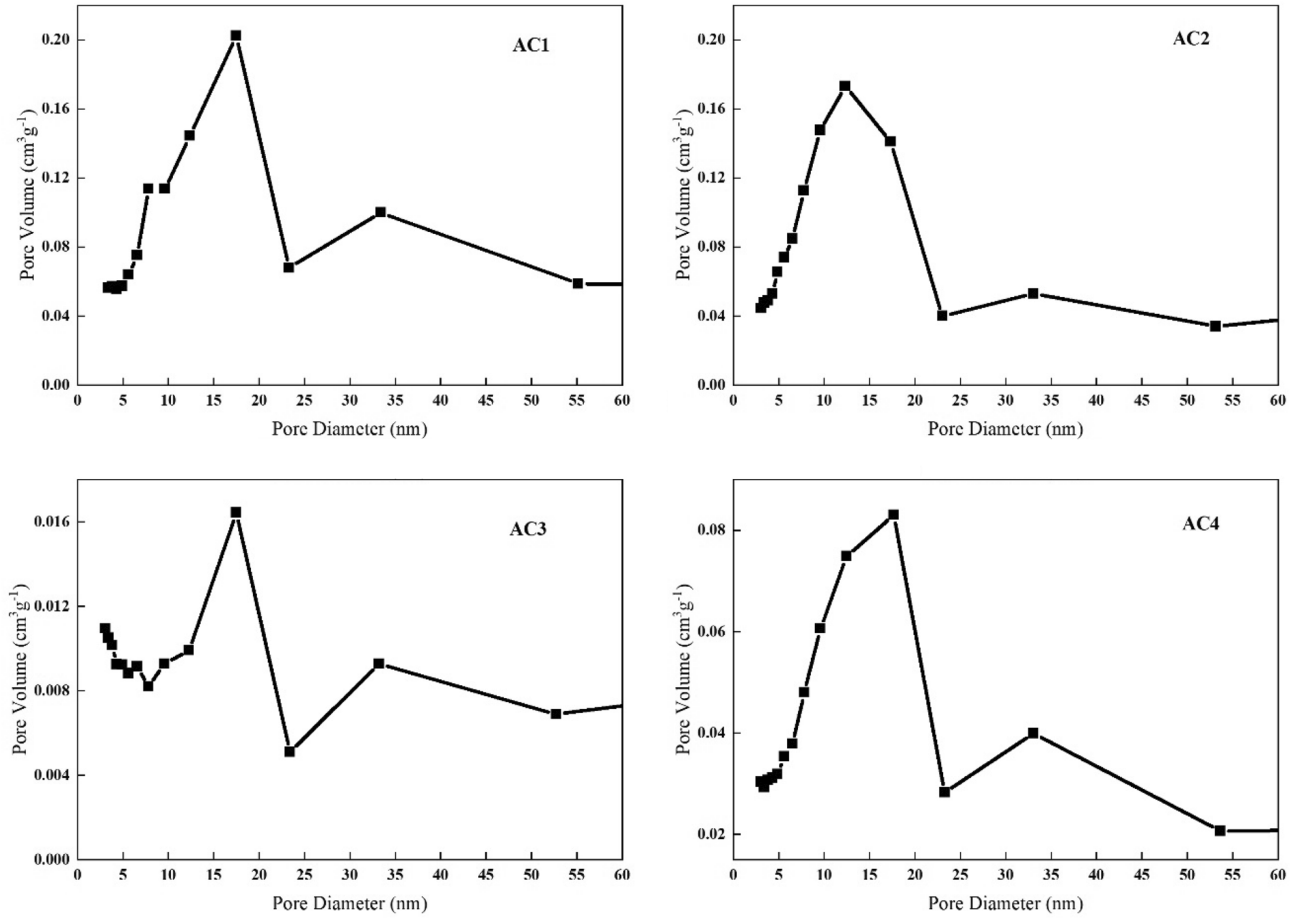
Pore size distribution of four activated carbons.

The V_Tot_ and pore distribution of the four ACs are different. From the experimental data, AC1 has the largest V_Tot_ and the largest V_Mic_, AC3 has the smallest V_Tot_ and the smallest V_Mic_, AC2 has the larger V_Tot_ and V_Mic_ than AC4, but the adsorption effect of AC2 is not as good as that of AC4. Therefore, the influence of V_Tot_ and pore distribution on adsorption capacity cannot be clearly obtained. However, studies have shown that the most effective pore size of adsorbent is 1.76 ~ 6 times of the molecular size of adsorbent^23^. Chl a and Chl b differ in only one substituent, the distance between particular extreme atoms of Chl a molecule is about 1.256 nm in one direction and 1.104 nm in another one^24^. Therefore, the pore size suitable for Chl a and Chl b adsorption is about 2–8 nm. Saleem et al.^14^ also showed that the size of dyes and humic acids (1.5 ~ 3.0 nm) was conducive to their adsorption in mesoporous pores. For adsorbents with molecular diameters of about 0.5 nm, microporous AC is usually sought as the primary adsorbent. The molecular sieve effect may greatly limit the adsorption, and the different pore size distribution may be one of the reasons for the different adsorption capacity of various ACs. The pore size distribution of four ACs is shown in Fig. 4.

**Figure 5:**
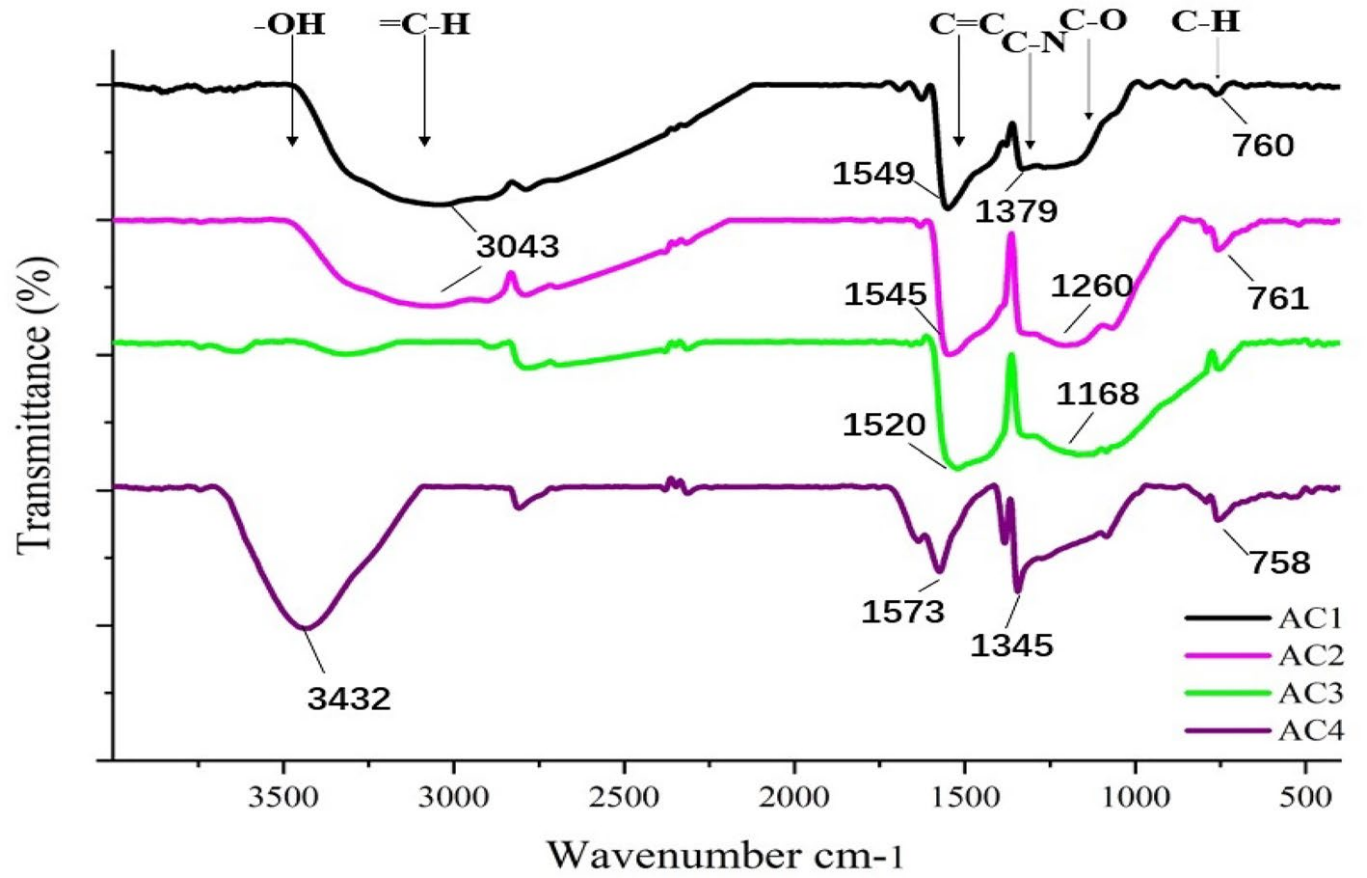
The FTIR spectra of four activated carbons.

Many chemical modifications have been used to increase the density of functional groups on the AC surface emphasizing the importance of functional groups interacting with polar substances in solution^25^. Figure 5 shows the FTIR spectra of four ACs and showed that the surfaces of all AC can act as donors and receptors for interaction, which is helpful in enhancing chlorophyll adsorption^20^. The peaks at 3319 cm^−1^ of AC3 and 3432 cm^−1^ of AC4 indicate the stretching vibration of –OH. The peak around 3000 cm^−1^ is probably the stretching vibration peak of the C–H bond on the benzene ring. The peak at around 1540 cm^−1^ is due to the aromatic C=C tensile vibration. The peak of 1000–1250 cm^−1^ may be the stretching vibration peak of C–O. The 1379 cm^−1^ peak of AC1 and 1345 cm^−1^ peak of AC4 indicate the possibility of C–N stretching vibration^21^.

**Figure 6:**
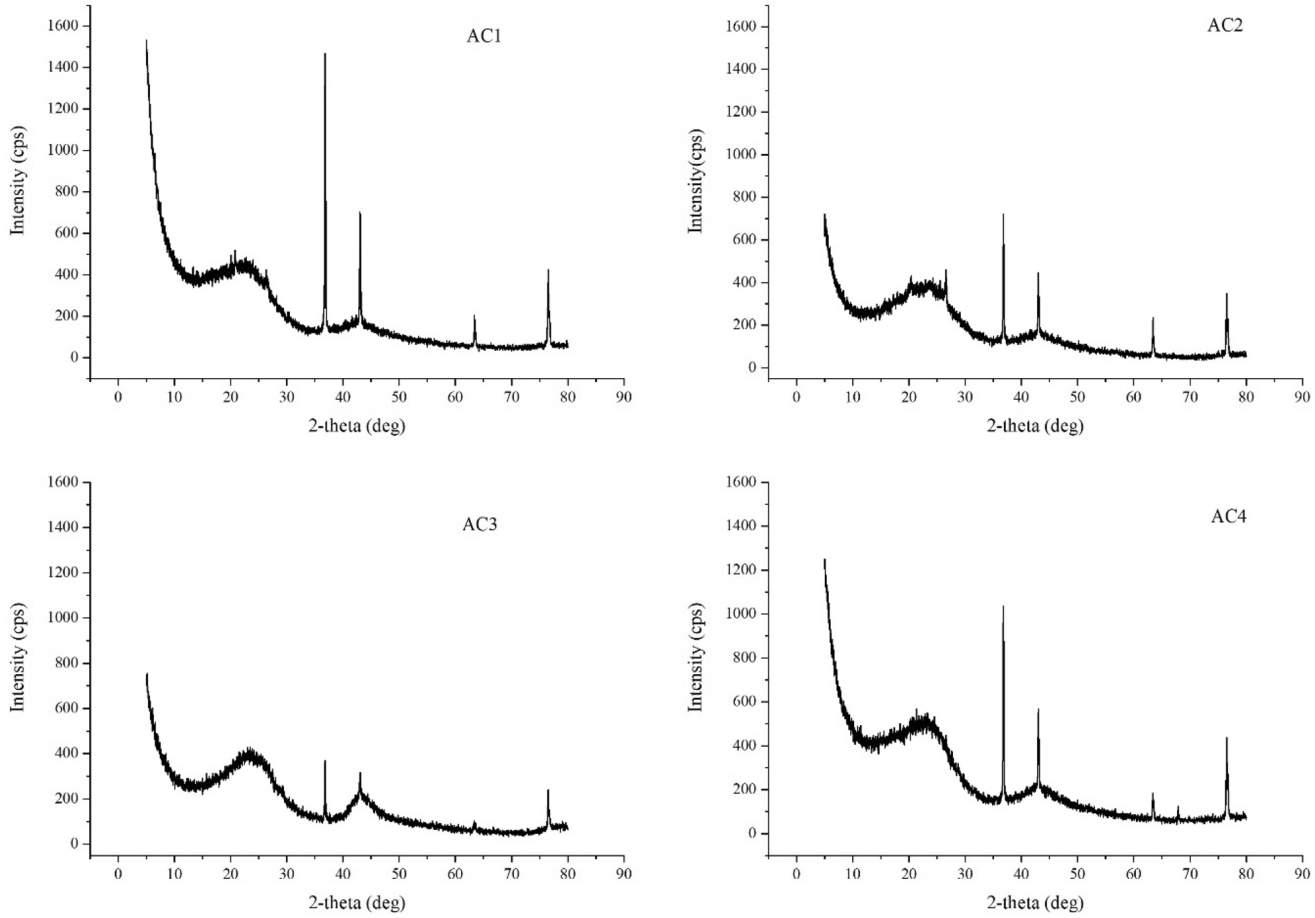
X-ray diffraction patterns of four activated carbons.

The diffraction spectrum of amorphous matter is one with two peaks near 24° and 42°. Crystalline material will appear a set of sharp diffraction peaks, the sharper the peak, the higher the crystallinity^26^. Figure 6 shows that the four ACs all have the properties of crystalline substances, and the crystallinity in descending order is AC1 > AC4 > AC2 > AC3.

### Adsorption isotherms

**Figure 7:**
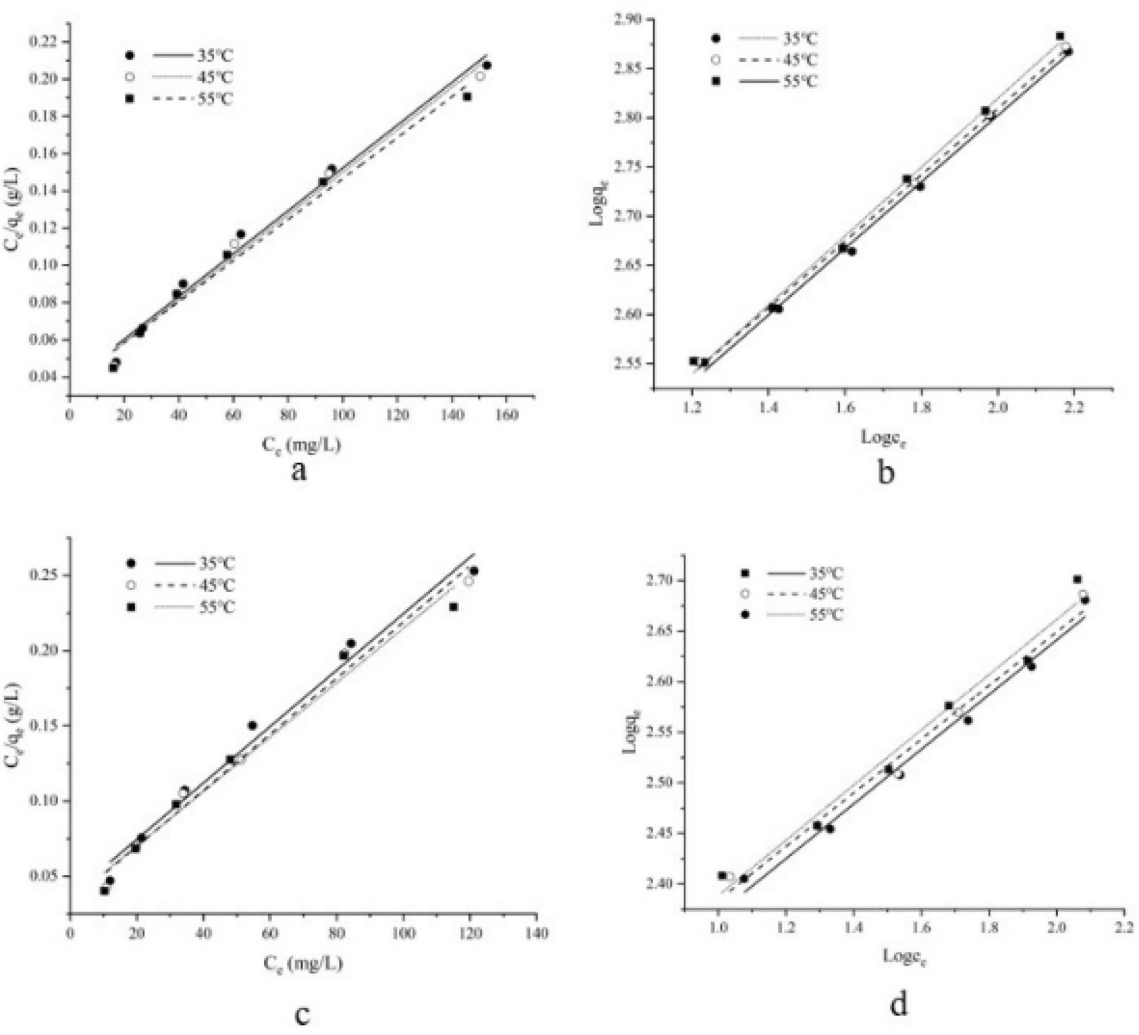
Langmuir and Freundlich models of Chl a and Chl b of AC1. (**a**) Langmuir model of Chl a; (**b**) Freundlich model of Chl a; (**c**) Langmuir model of Chl b; (**d**) Freundlich models of Chl b.

Langmuir model and Freundlich model are two kinds of adsorption isotherm models commonly used at present. According to the Langmuir model, the pollutants only covered the adsorbent in the form of a single molecular layer, while in Freundlich model, the pollutants covered the adsorbent through multiple layers^26^. The Langmuir and Freundlich adsorption isotherms and parameters of Chl a and Chl b adsorbed on AC1 are shown in Fig. 7, Tables 2 and 3.

**Table 2:** Adsorption isotherm model parameters of Chl a on AC1.

Temperatures ( C)	Langmuir adsorption isotherm model parameters				Freundlich adsorption isotherm model parameters		
	*K*_*L*_ (L mg^−1^)	*q*_*e*_ (mg g^−1^)	*R*_*L*_ (10^–5^)	*R*^*2*^	*K*_*F*_ (L g^−1^)	1/*n*	*R*^2^
35	31.456	869.565	9.104	0.988	131.759	0.337	0.997
45	32.452	877.193	8.823	0.988	133.693	0.338	0.998
55	33.127	909.090	8.642	0.985	136.386	0.350	0.996

**Table 3:** Adsorption isotherm model parameters of Chl b on AC1.

Temperatures ( C)	Langmuir adsorption isotherm model parameters				Freundlich adsorption isotherm model parameters		
	*K*_*L*_ (L mg^−1^)	*q*_*e*_ (mg g^−1^)	*R*_*L*_ (10^–5^)	*R*^*2*^	*K*_*F*_ (L g^−1^)	1/*n*	*R*^*2*^
35	17.527	531.915	2.229	0.983	125.930	0.265	0.987
45	18.724	537.634	2.144	0.986	130.458	0.270	0.985
55	19.654	552.486	2.042	0.978	131.374	0.273	0.977

At different temperatures, the *R*^2^ (ranging from 0.985 to 0.988) of Langmuir adsorption isotherms is relatively high; the *R*^*2*^ (ranging from 0.996 to 0.998) of Freundlich adsorption isotherm models can well fit the adsorption processes, both together indicated that the adsorption process of Chl a and Chl b on AC1 may be a complex adsorption process of single-layer and multilayer adsorption. The equilibrium parameter *R*_L_ of Langmuir model is close to 0 indicating that the adsorption process may be non-reversible adsorption.

In addition, with the increase of temperature, the *K*_F_ of Freundlich model showed a upward trend, and the adsorption performance also increased slightly. The constant 1/*n* ranges from 0.1 to 1, indicating that the adsorption is easy to carry out^26^.

The thermodynamic parameters of adsorption of Chl a and Chl b by AC1 are shown in Table 4. ∆*G*^0^ is all negative and the absolute value increases with the increase of temperature, indicating that the adsorption process is spontaneous, and rising temperature is conducive to the adsorption process. The positive value of ∆*H*^0^ indicates that the adsorption of Chl a and Chl b by AC1 is an endothermic reaction. ∆*S*^0^ is positive, indicating that the adsorption process is an affinity adsorption, and the adsorption process is an entropy increasing process.

**Table 4:** Thermodynamic parameters for Chlorophyll a and chlorophyll b adsorption process by activated carbon 1.

*T*(K)	*∆G*^*0*^ (KJ mol^−1^)	*∆H*^*0*^ (KJ mol^−1^)	*∆S*^*0*^ (J (mol*K)^−1^)
Chl a			
308	− 4.99	26.84	101.69
318	− 5.04		
328	− 6.37		
Chl b			
308	− 4.57	23.56	89.83
318	− 4.61		
328	− 5.78		

**Figure 8:**
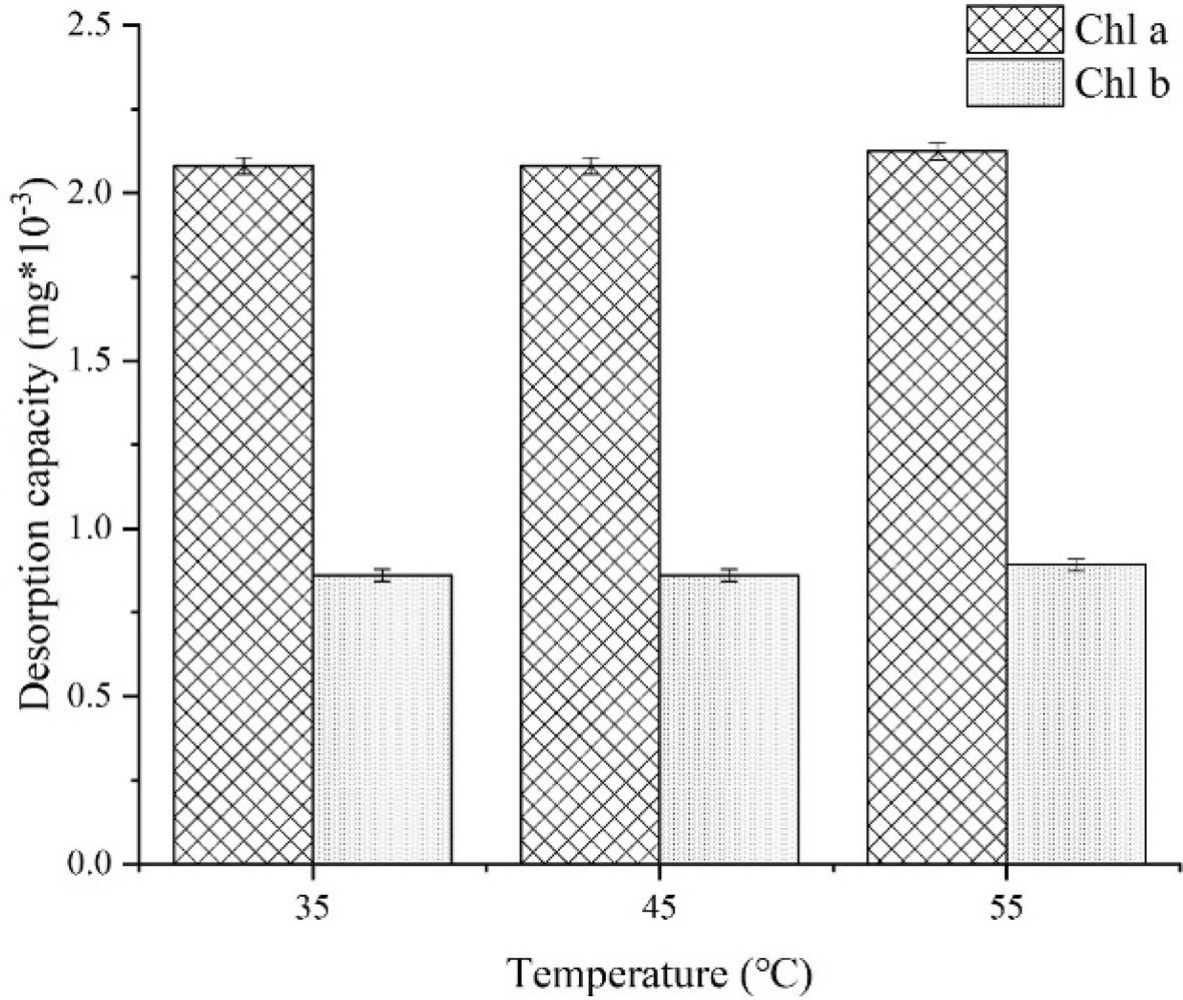
Desorption capacity of used AC1 in 90% ethanol solution.

The AC1 used was stirred in a clean 90% ethanol solution for 2 h at 35 °C, 45 °C and 55 °C to determine whether the adsorption was reversible. It was found that only trace amounts of Chl a and Chl b were detected in the supernatant (Fig. 8), indicating that the adsorption was indeed irreversible adsorption.

### Kinetic models

**Figure 9:**
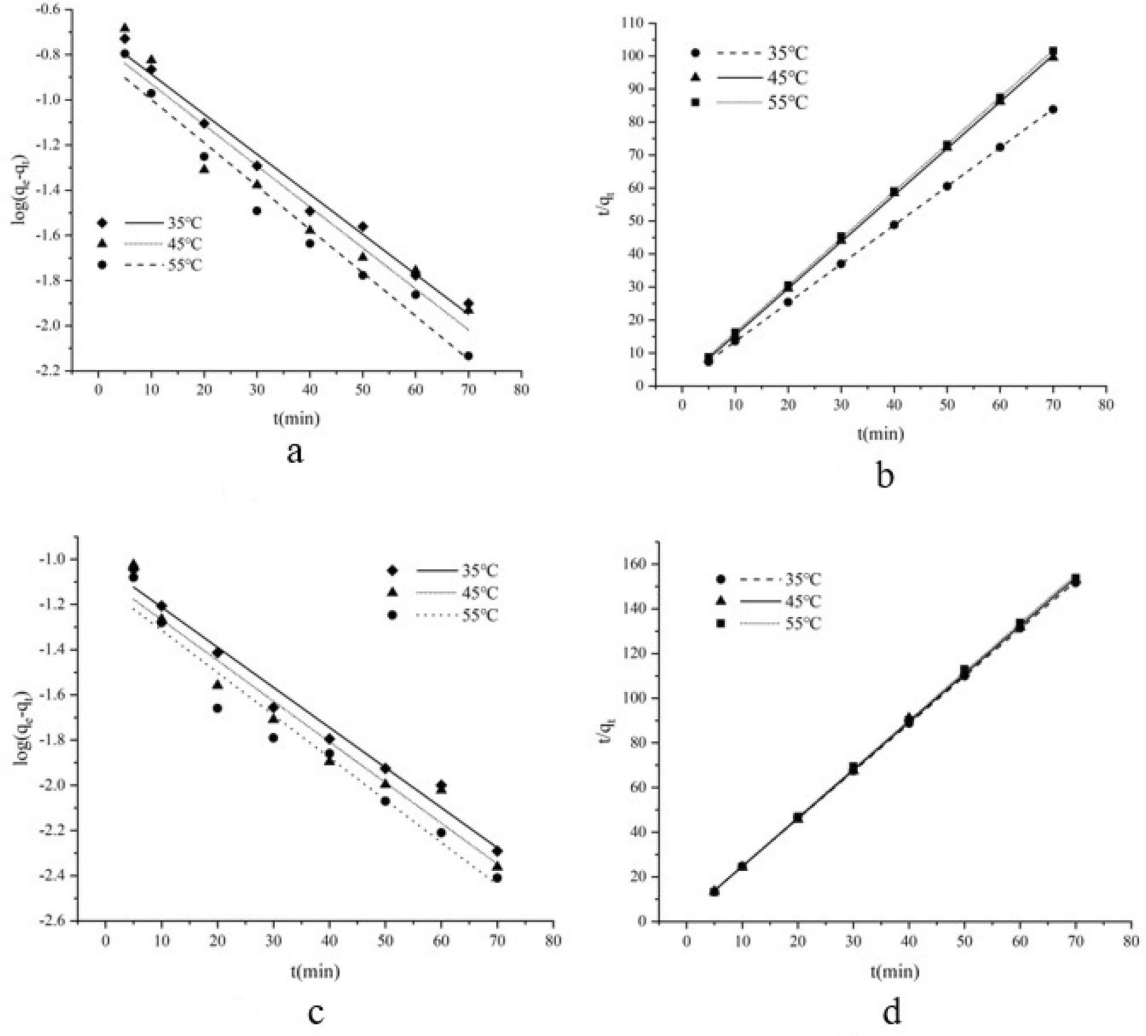
The pseudo-first-order and pseudo-second-order model of Chl a and Chl b adsorbed on AC1. (**a**) pseudo-first-order of Chl a; (**b**) pseudo-second-order of Chl a; (**c**) pseudo-first-order of Chl b; (**d**) pseudo-second-order of Chl b.

The pseudo-first-order and pseudo-second-order kinetic models proposed by Lagergren and Ho-McKay^27,28^, respectively, were employed to demonstrate the adsorption kinetics at different temperatures. For both Chl a and Chl b, the higher *R*^2^ indicates the better applicability of the pseudo-second-order model at all temperatures (*R*^2^ > 0.999) (Fig. 9 and Table 5). The pseudo-first-order equation is just applicable to describe the initial stage of kinetic adsorption and the adsorption is non-chemical unstable adsorption, while the pseudo-second-order equation is a hypothesis based on chemical adsorption and can fully reflect the adsorption process^29^. Therefore, the adsorption of Chl a and Chl b on AC1 is mainly based on chemisorption reaction.

**Table 5:** Pseudo-first order, pseudo-second order kinetics and intra-particle diffusion model parameters and correlation coefficients of Chl a and Chl b on AC1.

Model	Model parameter	Temperatures (°C)		
		35	45	55
Chl a pseudo-first-order model	*K*_1_	0.041	0.042	0.042
	*q*_*e*_	0.156	0.178	0.203
	*R*^2^	0.972	0.968	0.921
Chl a pseudo-second-order model	*K*_2_	0.794	0.991	1.536
	*q*_*e*_	0.702	0.747	0.851
	*R*^2^	1.000	0.999	1.000
Chl b pseudo-first-order model	*K*_1_	0.041	0.041	0.043
	*q*_*e*_	0.075	0.082	0.093
	*R*^2^	0.978	0.945	0.878
Chl b pseudo-second-order model	*K*_2_	2.128	4.478	5.091
	*q*_*e*_	0.303	0.323	0.470
	*R*^2^	1.000	0.999	1.000
Chl a intra-particle diffusion kinetic model	*K*_*p*_ (10^–1^)	0.062	0.069	0.072
	*C*	0.638	0.643	0.645
	*R*^2^	0.964	0.975	0.937
Chl b intra-particle diffusion kinetic model	*K*_*p*_ (10^–1^)	0.095	0.095	0.107
	*C*	0.376	0.377	0.380
	*R*^2^	0.950	0.972	0.990

### Intra-particle diffusion model

**Figure 10:**
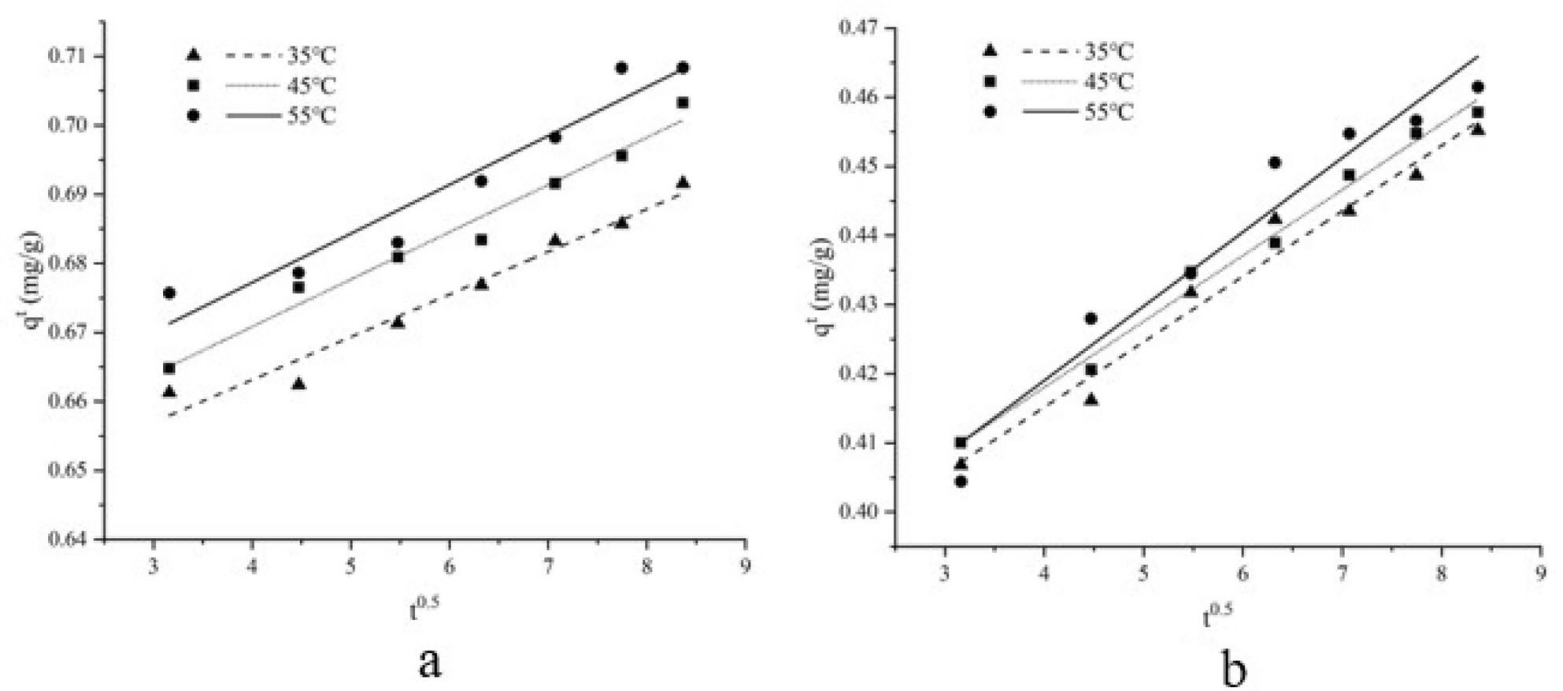
The intra-particle diffusion model of Chl a (**a**) and Chl b (**b**) adsorbed on AC1.

The adsorption mechanism was studied by using the model of intra particle diffusion proposed by Weber and Morris^30^. As shown in Fig. 10, the Chl a and Chl b adsorbed quality (*q*_*t*_) are linear to the square root curve of the contact time at all temperatures. The* R*^2^ (ranging from 0.937 to 0.990) (Table 5) of intra-particle diffusion model is relatively high, which implied intra-particle diffusion may be a rate-limiting step^31^. The lines in the model do not pass through the origin, suggesting that other mechanisms of mass transfer are occurring at the same time as the diffusion within the particle. In addition, with the increase of temperature, the value of boundary thickness (C) increased, indicating that the increase of temperature in the adsorption process may lead to the transformation of adsorption mechanism from diffusion and transfer to boundary layer film.

All the models studied in this paper are linear models. Compared with nonlinear models, linear models have the advantages of simple fitting, convenient fitting and fast fitting, but sometimes it will affect the accuracy of the model. For the sake of the intuitiveness of the data, we still use a linear model, which may be a weakness of this study. In future studies, it is necessary to further study the difference between linear and nonlinear fitting of AC to chl a and chl b adsorption models.

### π–π interaction

Π–π interaction refers to the interaction between the π electron in the carbon adsorbent and the π electron in the aromatic ring of the adsorbates^32^. Both Chl a and Chl b contain four nitrogen; Nitrogen usually act as strong electron acceptors. AC is generally considered to have a hexagonal structure formed by covalent bonds with the large π-bond structural properties of aromatic rings. Therefore, strong π–π interaction between AC1 and Chl a and Chl b is possible.

**Figure 11:**
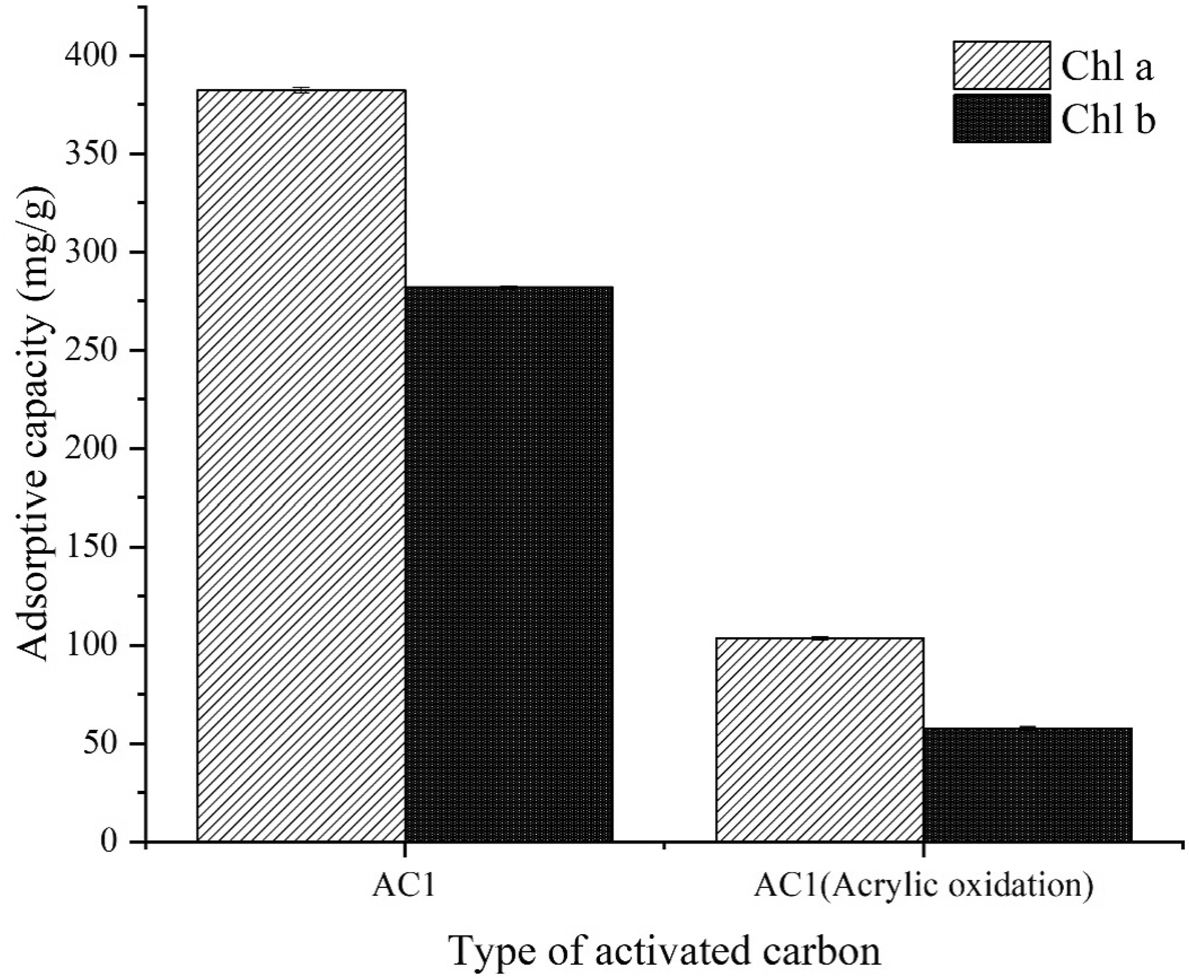
Effect of oxidation of acrylic acid on the removal of chlorophyll a and chlorophyll b by AC1 at 55 °C.

Acrylic oxidation experiments were used to determine the existence of π–π interaction. After adding oxygen groups to the aromatic ring structure of AC1, the adsorption rate decreased sharply (Fig. 11), which suggests that π–π interaction plays a vital role in the removal of Chl a and Chl b. The importance of π–π interaction has also been emphasized in the study of using AC to remove polycyclic aromatic hydrocarbons such as methylene green^33^, sulfamethoxazole^34^ and naphthalene^35^. In addition, FTIR revealed the presence of nitrogen on the AC1 surface, and the presence of polycyclic aromatic hydrocarbons in Chl a and Chl b, which may provide another part of the π–π interaction.

## Conclusion

The microstructures of four ACs were first characterized in order to reveal the internal factors for the difference in decolorization properties of Chl a and Chl b. The results show that the adsorption effect of AC on target adsorbent is affected by many factors. The S_BET_ of the four ACs from large to small is: AC1 (2525.68 m^2^ g^−1^) > AC2 (2058.83 m^2^ g^−1^) > AC4 (1639.88 m^2^ g^−1^) > AC3 (948.23 m^2^ g^−1^), however, adsorption effect of four ACs is: AC1 > AC4 > AC2 > AC3. This suggests that the specific surface area may be dominant but not decisive in the adsorption process. All the four ACs N_2_ adsorption–desorption isotherms show type IV isotherms with H4 hysteresis curves, which means that the four adsorbents have micropores and mesoporous. No strong correlation between pore volume, pore distribution and adsorption properties was observed in this study. FTIR shows that AC1 and AC4 contain nitrogen, which may enhance their adsorption of Chl a and Chl b. XRD results show that the four ACs have the properties of crystalline materials, and the crystallinity is AC1 > AC4 > AC2 > AC3.

AC1 has the best adsorption effect, so the adsorption isotherm, adsorption kinetics and adsorption mechanism were further studied on AC1. Langmuir model (*R*^2^ > 0.978) and Freundlich model (*R*^2^ > 0.977) indicated that the adsorption of Chl a and Chl b by AC1 may be a complex adsorption process of single layer adsorption and multilayer adsorption. The pseudo-second-order kinetic model (*R*^2^ > 0.999) indicated that AC1 adsorption was a chemisorption. The practicability of intra-particle diffusion model (*R*^2^ > 0.937) shows that intra-particle diffusion maybe a rate-limiting step. Oxidation experiments with acrylic acid show that π–π interaction is the key to the adsorption of Chl a and Chl b on AC1.

In conclusion, this study can not only provide reference for andrographolide manufacturers to screen AC for the removal of Chl a and Chl b, but also provide help for AC manufacturers to produce AC for the removal of Chl a and Chl b.

## Data Availability

The data generated and analyzed during this study are available from the corresponding author upon reasonable request.
