# Author Correction: Effect of activated carbon microstructure and adsorption mechanism on the efficient removal of chlorophyll a and chlorophyll b from *Andrographis paniculata* extract

**DOI:** 10.1038/s41598-024-55393-y

**Published:** 2024-03-08

**Authors:** Di Liang, Bao-yu Ji, Yun Wang, Xia Li, Wen-Yuan Gao

**Affiliations:** 1 Tianjin Key Laboratory for Advanced Drug Delivery & High-Efficiency, School of Pharmaceutical Science and Technology, Tianjin University, No. 92, Weijin Road, Nankai District, Tianjin, 300193 China; 2 College of Pharmacy, Henan University of Chinese Medicine, Zhengzhou, 450046 Henan China; 3 College of Pharmacy, Qinghai Minzu University, Qinghai, 810007 China

Correction to: *Scientific Reports* 10.1038/s41598-023-42011-6, published online 11 December 2023

In the original version of this Article, the equal contribution status of authors Di Liang and Bao-yu Ji was inadvertently omitted.

The original Article has been corrected.

